# P-920. Free-living Ameba Infections Diagnosed by Cell-free DNA Blood Testing – United States, 2018-2023

**DOI:** 10.1093/ofid/ofae631.1111

**Published:** 2025-01-29

**Authors:** Julia C Haston, Erin Imada, Shantanu Roy, Elizabeth Davlantes, Amy Freeland, Ibne Ali

**Affiliations:** Centers for Disease Control and Prevention, Atlanta, Georgia; Centers for Disease Control and Prevention, Atlanta, Georgia; Centers for Disease Control and Prevention, Atlanta, Georgia; Centers for Disease Control and Prevention, Atlanta, Georgia; Centers for Disease Control and Prevention, Atlanta, Georgia; Centers for Disease Control and Prevention, Atlanta, Georgia

## Abstract

**Background:**

Free-living amebae (FLA) including *Acanthamoeba* spp., *Balamuthia mandrillaris,* and *Naegleria fowleri* live in soil and water worldwide and cause severe infections in humans. Approximately 15 people in the United States are infected by FLA each year; most do not survive. Early diagnosis and treatment improve outcomes. Targeted testing options for FLA require tissue or cerebrospinal fluid (CSF) and are offered at < 5 laboratories in the U.S., making it challenging to diagnose infections quickly and non-invasively. Detection of FLA infections via cell-free DNA testing of blood (Karius Test®) has increased in recent years.

**Methods:**

The Centers for Disease Control and Prevention (CDC) maintains a database of confirmed FLA cases reported through the CDC FLA Clinical Consultation Service or national surveillance efforts. This database was used to identify and describe cases in which a Karius Test® first detected an FLA infection.

**Results:**

In 2018-2023, 9 U.S. FLA infections were diagnosed with Karius Test® and reported to CDC, including 4 *Acanthamoeba*, 4 *Balamuthia*, and 1 *Naegleria* case. These represent 10% of FLA cases reported to CDC in this timeframe (9/93). Four of these cases were reported in 2023 (22% of FLA cases in 2023), an increase over previous years. FLA had not been suspected in 8/9 cases before receiving results. Eight patients presented with meningitis or encephalitis; 1 had only skin findings. FLA diagnoses were subsequently confirmed by other methods in 7/7 cases for which tissue or CSF was available. Duration from hospitalization or symptom onset to Karius Test® result was 5-14 days. One patient was diagnosed postmortem; the others were initiated on anti-amebic therapy. No patients ultimately survived, though 3 patients were treated for several weeks.

**Conclusion:**

Cell-free DNA testing (Karius Test®) of blood is increasingly being used to diagnose FLA infections. Nearly all patients described were diagnosed by Karius Tests® before FLA infections had been suspected. Early diagnosis can allow early treatment, inform clinical decision-making, and contribute to the further understanding of FLA infections through surveillance. In patients with suspected FLA infections or encephalitis of unknown etiology, Karius Test® may be useful, particularly when tissue or CSF is not available.

**Disclosures:**

**All Authors**: No reported disclosures

